# EMOTION REGULATION THROUGH MUSIC AND MINDFULNESS ARE RELATED TO POSITIVE SOLITUDE

**DOI:** 10.1093/geroni/igad104.1057

**Published:** 2023-12-21

**Authors:** Ehud Bodner, Yuval Palgi, Noa Bachman

**Affiliations:** Bar Ilan University, Tel Aviv, Tel Aviv, Israel; University of Haifa, Haifa, Hefa, Israel; University of Haifa, Haifa, Hefa, Israel

## Abstract

Mindfulness and emotion regulation through music listening are skills that share some attributes with the skill of positive solitude (PS; defined as an inner choice to dedicate time to a meaningful, enjoyable activity or experience managed by oneself, with or without the presence of others). Nevertheless, little is known about their relationship with PS in the second half of life. Hence, we recruited a convenience sample of community-dwelling adults in the second half of life (N = 123; M = 68.63, SD = 10.99), who completed self-report measures of demographics, emotion regulation through music, mindfulness, and PS. A hierarchical linear regression demonstrated significant positive associations between emotion regulation through music listening and PS, and between mindfulness and PS. Moreover, age moderated the relationship between mindfulness and PS. This relationship was found to be positive and significant only among older adults. These findings support the study’s hypotheses and emphasize the contribution of the current research to developmental research on PS in the second half of life.

